# Allyl isothiocyanate regulates lysine acetylation and methylation marks in an experimental model of malignant melanoma

**DOI:** 10.1007/s00394-019-01925-6

**Published:** 2019-02-14

**Authors:** Melina Mitsiogianni, Theodora Mantso, Dimitrios T. Trafalis, H. P. Vasantha Rupasinghe, Vasilis Zoumpourlis, Rodrigo Franco, Sotiris Botaitis, Aglaia Pappa, Mihalis I. Panayiotidis

**Affiliations:** 1 Department of Applied Sciences, Translational Biosciences Group, Northumbria University, Ellison Building, Newcastle Upon Tyne, NE1 8ST UK; 2 Laboratory of Pharmacology, Clinical Pharmacology Unit, Medical School, National and Kapodistrian University of Athens, 11527 Athens, Greece; 3 Department of Plant, Food and Environmental Sciences, Faculty of Agriculture, Dalhousie University, Halifax, NS B3H 4R2 Canada; 4 Department of Pathology, Faculty of Medicine, Dalhousie University, Halifax, NS B3H 4R2 Canada; 5 Biomedical Applications Unit, Institute of Biology, Medicinal Chemistry and Biotechnology, National Hellenic Research Foundation, 11635 Athens, Greece; 6 Redox Biology Centre, University of Nebraska, Lincoln, 68583 USA; 7 Department of Veterinary Medicine and Biomedical Sciences, University of Nebraska, Lincoln, 68583 USA; 8 Second Department of Surgery, Medical School, Democritus University of Thrace, 68100 Alexandroupolis, Greece; 9 Department of Molecular Biology and Genetics, Democritus University of Thrace, 68100 Alexandroupolis, Greece

**Keywords:** Allyl isothiocyanate, Skin cancer, Acetyl transferases, Deacetylases, Methyl transferases, Histone acetylation, Histone methylation

## Abstract

**Objective(s):**

Isothiocyanates (ITCs) are biologically active plant secondary metabolites capable of mediating various biological effects including modulation of the epigenome. Our aim was to characterize the effect of allyl isothiocyanate (AITC) on lysine acetylation and methylation marks as a potential epigenetic-induced anti-melanoma strategy.

**Methods:**

Our malignant melanoma model consisted of (1) human (A375) and murine (B16-F10) malignant melanoma as well as of human; (2) brain (VMM1) and lymph node (Hs 294T) metastatic melanoma; (3) non-melanoma epidermoid carcinoma (A431) and (4) immortalized keratinocyte (HaCaT) cells subjected to AITC. Cell viability, histone deacetylases (HDACs) and acetyltransferases (HATs) activities were evaluated by the Alamar blue, Epigenase HDAC Activity/Inhibition and EpiQuik HAT Activity/Inhibition assay kits, respectively, while their expression levels together with those of lysine acetylation and methylation marks by western immunoblotting. Finally, apoptotic gene expression was assessed by an RT-PCR-based gene expression profiling methodology.

**Results:**

AITC reduces cell viability, decreases HDACs and HATs activities and causes changes in protein expression levels of various HDACs, HATs, and histone methyl transferases (HMTs) all of which have a profound effect on specific lysine acetylation and methylation marks. Moreover, AITC regulates the expression of a number of genes participating in various apoptotic cascades thus indicating its involvement in apoptotic induction.

**Conclusions:**

AITC exerts a potent epigenetic effect suggesting its potential involvement as a promising epigenetic-induced bioactive for the treatment of malignant melanoma.

## Introduction

Melanoma is an aggressive and highly metastatic type of skin cancer with significantly increasing incidence rates over the past few years [1, 2]. Thus, the design of new approaches in disease prevention and treatment is of great importance. To this end, consumption of cruciferous vegetables has been strongly associated with reduced risk of cancer development particularly because of their rich content in isothiocyanates (ITCs) [3]. These are compounds produced through hydrolysis of their precursor molecules, glucosinolates, by an enzyme called myrosinase which is activated after plant tissue disruption [4]. In general, ITCs are important nutraceutical agents capable of protecting against cancer development [5, 6] by a plurality of biological activities including modulation of detoxification enzymes, induction of apoptosis and cell cycle arrest and interaction with various signaling pathways [7–11]. With respect to melanoma, both in vitro and in vivo studies have shown that various ITCs can induce apoptosis and cell cycle arrest thus suppressing tumor growth [12–15]. Further evidence also supports the involvement of ITCs in gene regulation by reversing cancer-associated epigenetic marks at both DNA and histone levels [16–21].

In general, epigenetic mechanisms such as DNA methylation and histone modifications can act in a coordinative and complex manner resulting in conformational changes to chromatin, which regulate the genetic information by providing access to regulatory molecules, i.e., transcription factors, etc. [22]. More specifically, histone proteins can undergo various modifications in their N-terminus (including among others methylation and acetylation) that directly affect the state of chromatin structure. For instance, histone acetylation is regulated by the opposing action of two enzymes: histone deacetylases (HDACs) and histone acetyl transferases (HATs), while histone methylation is catalyzed by histone methyltransferases (HMTs). Generally, acetylation of histones leads to gene activation while histone methylation results in either the activation or suppression of genes based on the site of the specific modification [23–25].

In principle, HDACs de-acetylate lysine residues on histone tails and consequently lead to gene silencing. In addition, non-histone proteins are also substrates for these enzymes, an important aspect for their function in health and disease [26]. HDACs are at an equilibrium state with HATs (which acetylate their substrates) resulting in transcriptional activation, and it’s the interplay between them that controls acetylation status of their substrates [27, 28]. However, chromatin structure regulation is even more complex and affected by the cross-talk between acetylation and methylation enzymes [29]. Even though there is enough information about ITCs’ involvement in the epigenetic regulation of different cancers, there is only a limited number of studies investigating their ability to induce epigenetic responses in malignant melanoma cells [30, 31].

The aim of this study was to investigate the involvement of AITC as an epigenetic regulator capable of modulating specific lysine acetylation and methylation marks, on histones 3 (H3) and 4 (H4) and thus potentially regulating gene expression which could ultimately lead to inhibition of cell growth in malignant melanoma.

## Materials and methods

### Chemicals

Allyl isothiocyanate (AITC) was obtained from Sigma-Aldrich (St. Louis, MO, USA) dissolved in dimethyl sulfoxide (DMSO; Sigma-Aldrich) and stored at − 20 °C. Dulbecco modified Eagle medium (DMEM), trypsin, phosphate buffer saline (PBS), fetal bovine serum (FBS), l-glutamine and penicillin/streptomycin were obtained from Labtech International Ltd (East Sussex, UK). Resazurin sodium salt was supplied by Sigma-Aldrich. All chemicals were of analytical grade and purchased from Sigma-Aldrich, Applichem (Darmstadt, Germany) and Invitrogen (Carlsbad, CA, USA). Bovine Serum Albumin (BSA) was obtained from Biosera (Boussens, France). Protease and phosphatase inhibitor cocktails were obtained from Roche (Basel, Switzerland). Polyvinylidene difluoride (PVDF) membranes (0.45 and 0.2 µm) were purchased from Millipore (Bedford, MA, USA).

### Cell culture and exposure protocol to AITC

The A375 and A431 cell lines were purchased from Sigma-Aldrich while the HaCaT cell line was kindly provided by Dr Sharon Broby (Dermal Toxicology and Effects Group; Centre for Radiation, Chemical and Environmental Hazards; Public Health England, UK). In addition, the VMM1, Hs 294T and B16-F10 cell lines were obtained from LGC Standards (Middlesex, UK). The A375, A431, Hs 294T, HaCaT and B16-F10 cell lines were cultured in DMEM high glucose medium [10% FBS, 2 mM l-glutamine (4 mM l-glutamine for Hs 294T) and 1% penicillin/streptomycin]. Finally, the VMM1 cell line was cultured in RPMI-1640 high glucose medium (10% FBS, 2 mM l-glutamine and 1% penicillin/streptomycin). All cell lines were maintained in a humidified atmosphere at 37 °C and 5% CO_2_. AITC or vehicle was added as a single bolus concentration ranging between 2.5 and 50 µΜ for 24 h and 48 h.

### Determination of cell viability

All cell lines were seeded in 100 µl of the complete medium into 96-well plates and kept overnight in the incubator before they were exposed to AITC at various concentrations, for 24 h and 48 h. Cell viability was assessed by using the Alamar blue assay where, in brief, resazurin sodium salt was dissolved in PBS (1 mg/ml final concentration) and added in an amount equal to 1/10 of the volume in each well. After 4 h of incubation at 37 °C, absorbance was measured at 570 nm using 600 nm as a reference wavelength by using a Tecan Spark 10M plate reader (Männedorf, Switzerland).

### Exposure protocols to decitabine, panobinostat and anacardic acid

A375 cells were exposed to three different combination protocols involving AITC and either decitabine, panobinostat or anacardic acid. Each of the experimental exposure conditions were as follows: (1) co-treatment of AITC with either of the inhibitors over 48 h (Protocol 1); (2) pre-treatment with either of the inhibitors for 24 h followed by co-treatment with AITC and either inhibitor for an additional 48 h (Protocol 2) and (3) pre-treatment with either of the inhibitors for 24 h followed by treatment with AITC only for 48 h (Protocol 3). Decitabine and panobinostat were purchased from Selleckchem (Houston, TX, USA) while anacardic acid from Abcam (Cambridge, UK). Decitabine was used at 1–50 µM, panobinostat at 2.5–100 nM and anacardic acid at 5–150 µM concentration ranges. Stock solutions were prepared in DMSO at 20 mM (decitabine and panobinostat) and 25 mM (anacardic acid), respectively, and were stored at − 20 °C.

### Preparation of cell lysates and protein determination

A375 cells were plated in 100-mm dishes and cultured overnight at 37 °C. Next day, cells were treated with 10 µΜ of AITC for 48 h and then trypsinized, collected in micro-centrifuge tubes and washed twice with PBS. Nuclear and cytosolic lysates were obtained using the NE-PER Nuclear and Cytoplasmic Extraction Kit from Thermo Scientific (Waltham, MA, USA). Total histone extracts were obtained using the EpiQuik Total Histone Extraction Kit from Epigentek (Farmingdale, NY, USA). Protein content was determined by utilizing the BCA protein assay kit from Thermo Scientific (Waltham, MA, USA). All extraction and assay kits were used according to the manufacturer’s protocols. Protein extracts were stored at − 20 or − 80 °C (for estimation of HDAC/HAT activities) until usage.

### Determination of HDAC and HAT activities

The Epigenase HDAC Activity/Inhibition Direct Assay kit and the EpiQuik HAT Activity/Inhibition Assay kit were purchased from Epigentek (Farmingdale, NY, USA) and were used for the determination of total HDAC and HAT activity levels according to the manufacturer’s protocol. For HDAC determination, nuclear cell lysates were prepared and 10 µg of extracts were incubated with an acetylated substrate for 90 min at 37 °C. Similarly, for HAT determination, 10 µg of nuclear extracts were incubated with a histone substrate for 60 min at 37 °C. Optical density values were monitored at 450 nm with an optional reference wavelength of 655 nm using a Tecan Spark 10M plate reader (Männedorf, Switzerland).

### Western immunoblotting

Forty micrograms (40 µg) of cytoplasmic, 20 µg of nuclear and 15 µg of histone protein extracts were separated by SDS-polyacrylamide gels and transferred electrophoretically onto PVDF membranes (either 0.45 or 0.2 µm) using the mini-gel tank and mini-blot modules from Invitrogen (Carlsbad, CA, USA), respectively. The blots were then blocked in 5% non-fat milk powder in TBST buffer (50 mM Tris–HCl, 150 mM NaCl at pH 7.6 and 0.1% Tween-20) for 2 h at room temperature. After blocking, membranes were washed three times with TBST and incubated overnight at 4 °C, under agitation, with the appropriate primary antibody and according to the manufacturer’s protocol. Next day, membranes were incubated with the appropriate horseradish peroxidase-conjugated secondary antibody (mouse or rabbit at 1:1000) for 1 h at room temperature, under agitation, after being washed three times with TBST. After incubation with the secondary antibody, membranes were washed three times with TBST and labeled protein bands were detected by utilizing the SuperSignal West Pico PLUS Chemiluminescent Substrate from Thermo Scientific (Waltham, MA, USA) according to the manufacturer’s protocol. Protein bands were visualized with the use of the G:BOX Chemi XX6/XX9 gel imaging system (Syngene, Cambridge, UK).

### RNA extraction and determination of apoptotic gene profiling by RT-PCR-based microarrays

A375 cells were plated in 100-mm dishes, cultured overnight, exposed to either 10 µM AITC (treatment) or 0.1% DMSO (control) for 48 h, collected via trypsinization and then washed twice with cold PBS. Total RNA was extracted using the TRIzol reagent according to the manufacturer’s protocol (Invitrogen, Waltham, MA, USA). Quality and concentration of RNA were assessed by agarose gel electrophoresis and spectrophotometric analysis, respectively. Complimentary DNA (cDNA) was synthesized by using the SuperScript VILO cDNA synthesis kit (Invitrogen) according to the manufacturer’s protocol. Quantitative PCR (qPCR) was carried out by utilizing the TaqMan Array Human Apoptosis 96-well plates (Applied Biosystems, Carlsbad, CA, USA). TaqMan Universal master mix (2×) was added to an equal amount of diluted cDNA (5–50 ng per well in RNAase-free water) with 10 µl of the total mixture being added into each well. Real-time PCR (RT-PCR) was performed on a StepOne Plus RT-PCR instrument (Applied Biosystems, Carlsbad, CA, USA), whereas gene expression data were analyzed by the ΔΔCt method. Differences in apoptotic gene expression were indicated as fold change by using the DataAssist v3.01 software.

### Statistical analysis

In all sets of experiments, data were expressed as mean values ± SEM and comparisons were made between control and exposure (treatment) groups. Calculations were performed by using the Microsoft Office Excel 2016 software. Means were compared by one-way analysis of variance (one-way ANOVA) with Tukey’s test for multiple comparisons for viability assays and paired *t* test for HDAC/HAT activity assays and western immunoblotting densitometric data. SPSS v.22 software was used for statistical tests. A value of *p* < 0.05 was considered statistically significant.

## Results

### AITC suppresses cell viability in A375, Hs 294T and B16-F10 but not in VMM1, A431 and HaCaT cells

**Fig. 1:**
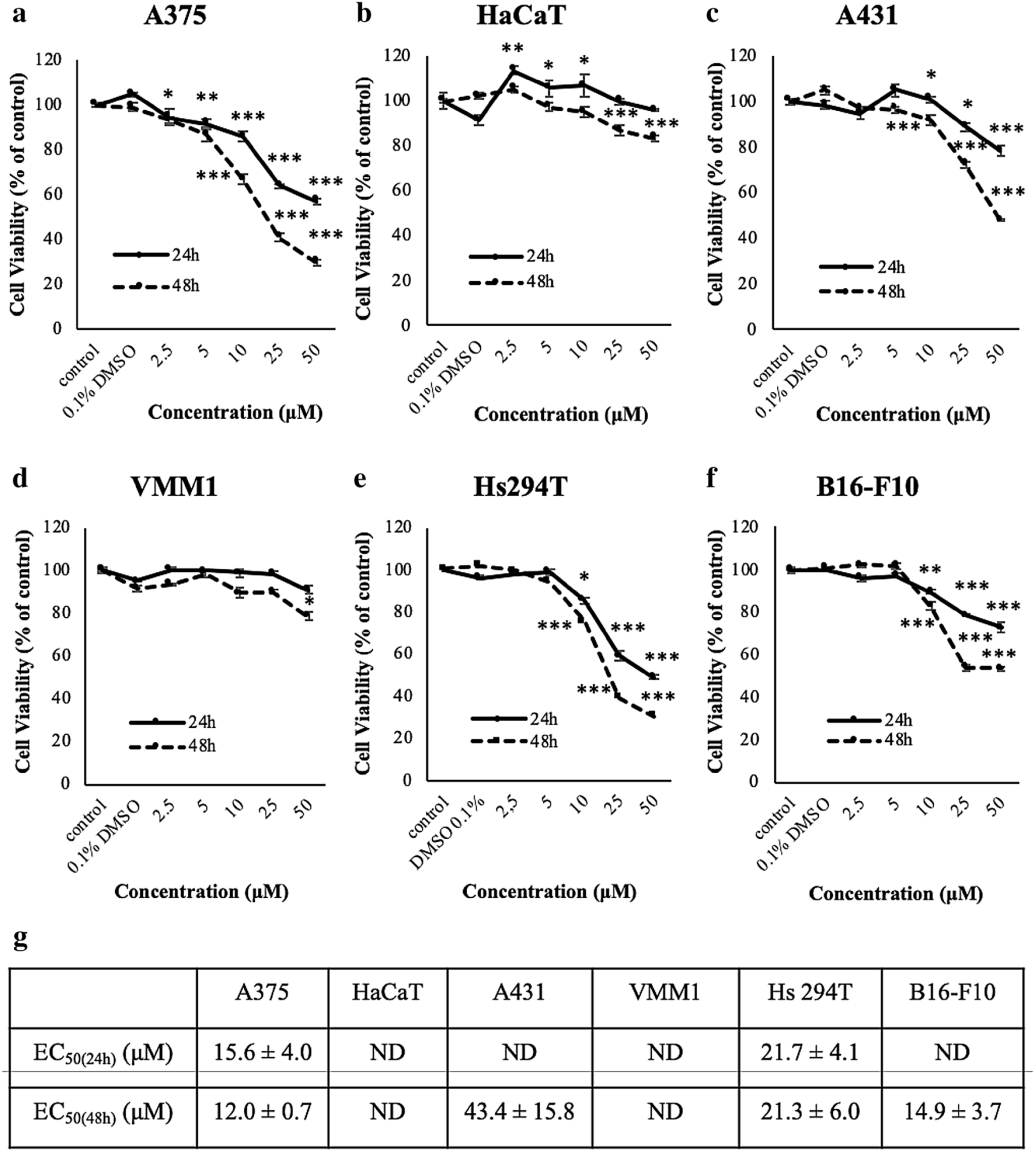
AITC-induced cytotoxicity in an in vitro model of malignant melanoma. The experimental model consisted of human: **a** malignant melanoma (A375); **b** keratinocyte (HaCaT); **c** non-melanoma epidermoid carcinoma (A431); **d** brain metastatic melanoma (VMM1) and **e** lymph node metastatic melanoma (Hs 294T) as well as **f** murine malignant melanoma (B16-F10) cells exposed to a single bolus concentration of AITC (2.5–50µΜ) at 24 and 48 h of exposure; **g** EC_50_ values were estimated for all cell lines at each exposure time point to AITC. Data are expressed as means ± SEM and are representative of three independent experiments. Statistical significance was set at *p < 0.05, **p < 0.01, ***p < 0.001 relative to corresponding (DMSO) controls. Finally, “ND” denotes “not determined”

To investigate the anti-melanoma effect of AITC, we utilized a malignant melanoma model consisting of human (A375) and murine (B16-F10) malignant melanoma cells as well as of human brain (VMM1) and lymph node (Hs 294T) metastatic melanoma cells in addition to non-melanoma epidermoid carcinoma (A431) and immortalized keratinocyte (HaCaT) cells, subjected to a range of AITC concentrations (2.5–50 µM) for 24 h and 48 h. Overall, it was shown that AITC reduced the viability of A375, Hs 294T and B16-F10 cells in a concentration- and time-dependent manner (Fig. 1a, e, f) compared to HaCaT, A431 and VMM1 cells (Fig. 1b–d). More specifically, in A375 cells, AITC decreased viability at 10 µM onwards as it did with Hs 294T and B16-F10 cells, respectively. On the contrary, at the same experimental conditions, the viability levels for HaCaT, A431 and VMM1 cells were minimally affected as they were shown to be more resistant. Based on the cytotoxicity profile of each cell line, a concentration of 10 µM over 48 h of exposure was chosen as optimum experimental conditions. Moreover, the use of A375 cell line was chosen in all experiments described herein on the basis of being the most sensitive one to the effects of AITC. Finally, the EC_50_ values for all cell lines were calculated (for both 24 and 48 h of AITC exposure) confirming that A375, Hs294T and B16-F10 cells were more sensitive to the cytotoxic effect of AITC compared to HaCaT, A431 and VNM1 ones (Fig. 1g).

### Exposure to AITC is not associated with DNA methylation levels in human malignant melanoma (A375) cells

**Fig. 2:**
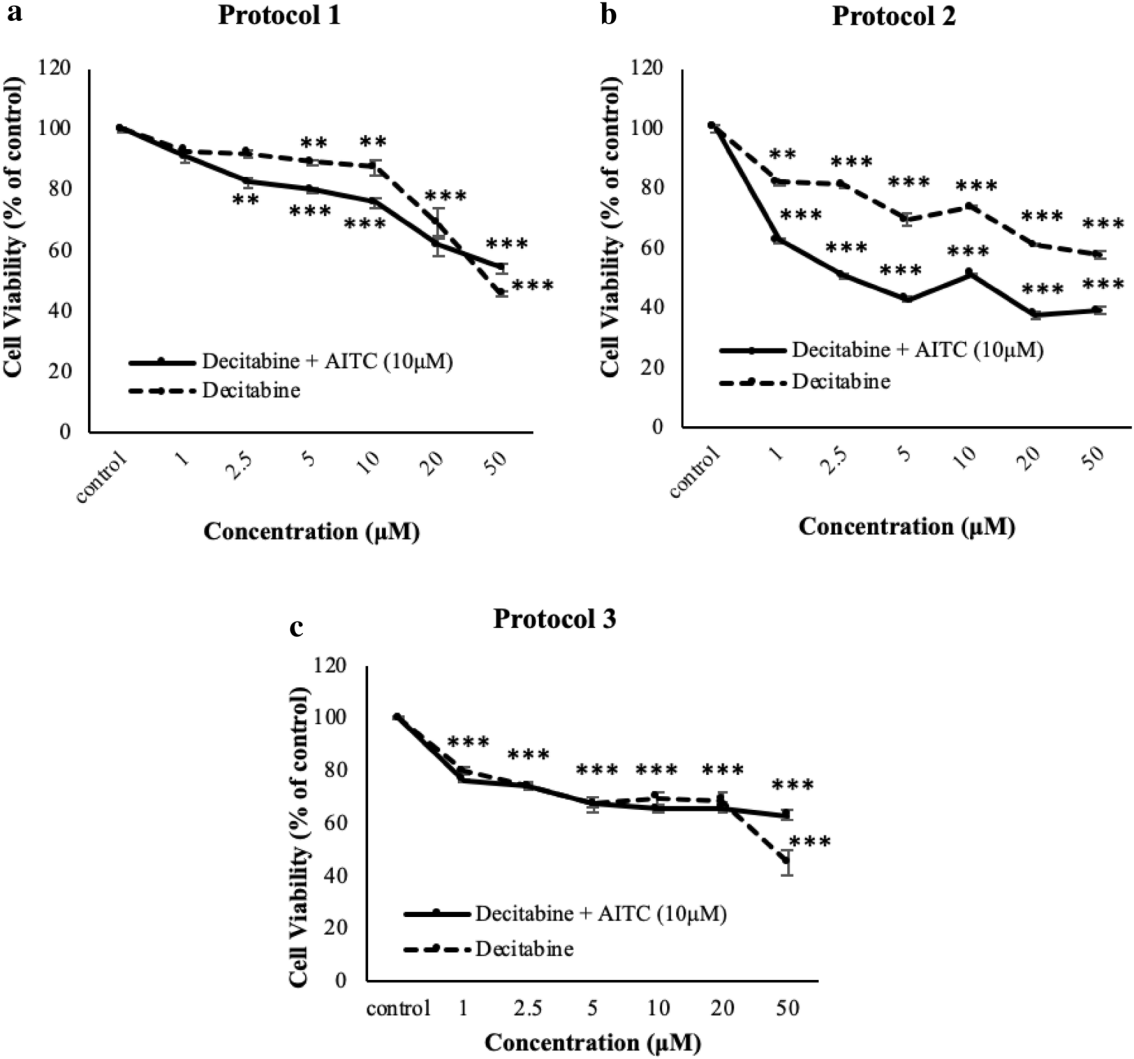
The effect of AITC on DNA methylation status in human malignant melanoma (A375) cells. A375 cells were exposed to AITC (10 µM) and decitabine under three experimental protocols (described in “Materials and methods”): **a** Protocol 1; **b** Protocol 2 and **c** Protocol 3. Data are expressed as means ± SEM and are representative of three independent experiments. Statistical significance was set at *p < 0.05, **p < 0.01, ***p < 0.001 relative to corresponding controls

In this set of experiments, we aimed to investigate if the observed AITC-induced decline in viability levels of A375 cells was associated with an elevation in DNA methylation status. For this reason, we utilized decitabine (a DNA methyltransferase inhibitor) at a range of concentrations (1–50 µM) under all three of the above-mentioned experimental protocols. According to our results, co-treatment of decitabine with AITC (under experimental conditions of protocol 2) led to a further reduction in viability levels when compared to AITC alone. The other two protocols did not cause a significant change in cell viability (Fig. 2a–c). In conclusion, it is evident that AITC-induced reduction in cell viability cannot be linked to an increased DNA methylation status as the co-treatment protocol did not reverse the cytotoxic effect of AITC in A375 cells.

### AITC reduces specific HDAC and HAT protein expression levels and diminishes specific histone H4 lysine acetylation marks in human malignant melanoma (A375) cells

**Fig. 3:**
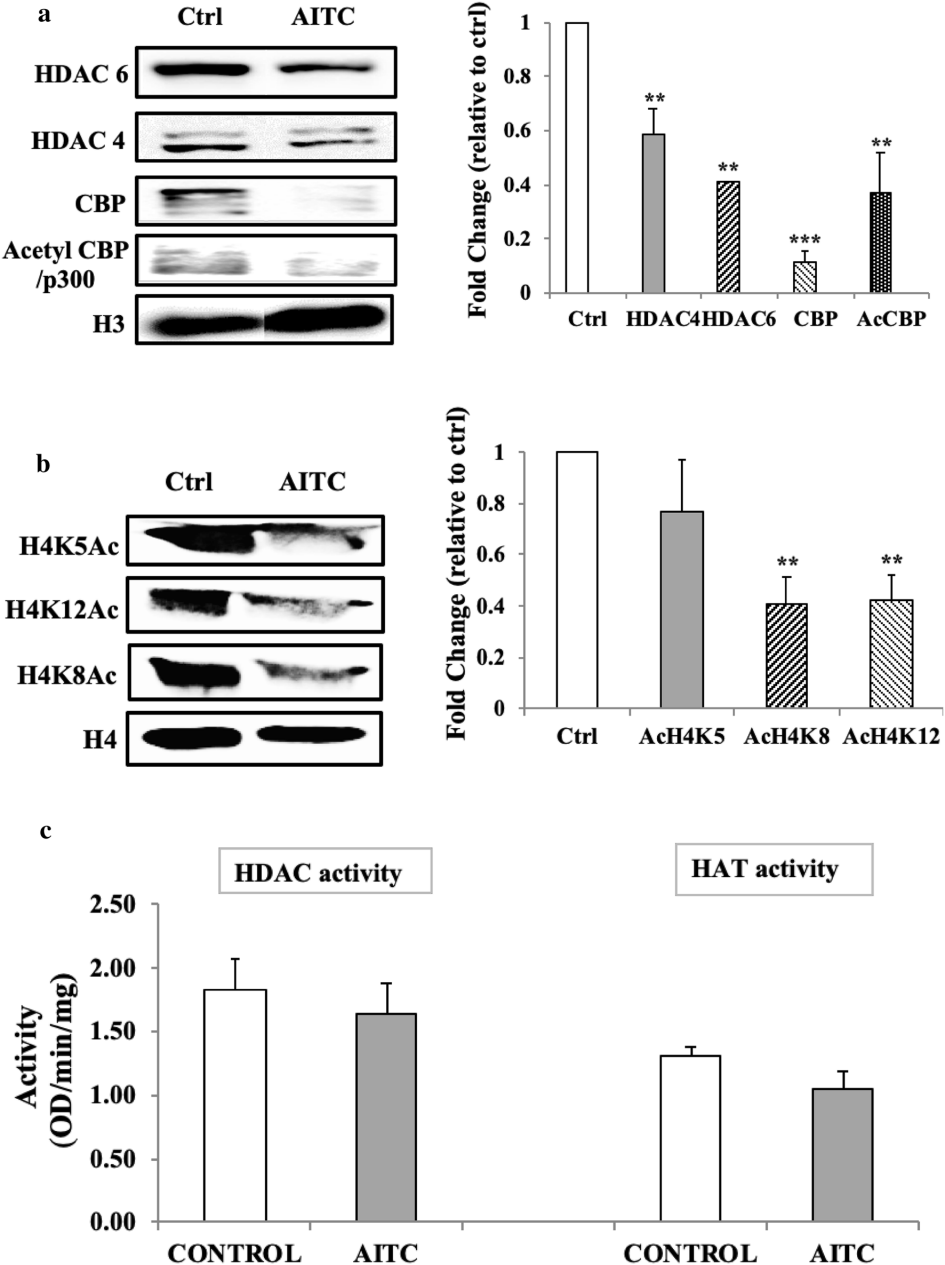
The effect of AITC on histone acetylation, deacetylation and specific H4 lysine acetylation marks in human malignant melanoma (A375) cells. A375 cells were exposed to 10 µM of AITC for 48 h. Western immunoblotting was used, in nuclear and histone extracts, in order to assess the expression levels of **a** HDACs 4 and 6 as well as those of HATs including CBP and Acetyl-CBP/p300; **b** the acetylation status of H4K5, H4K8 and H4K12. **c** Enzymatic activities of HDAC and HAT were evaluated using the Epigenase HDAC Activity/Inhibition Direct assay kit and the EpiQuik HAT Activity/Inhibition Assay Kit, respectively. In all experiments, data were normalized to the corresponding untreated control (Ctrl) and are representative of three independent experiments. Statistical significance was set at *p < 0.05, **p < 0.01, ***p < 0.001 relative to corresponding controls

Protein expression levels of various HDACs (e.g., 1, 2, 4, 6, and phospho HDAC 4/5/7) and HATs (e.g., CBP, Acetyl CBP/p300, PCAF, and GCN5L2) were evaluated in nuclear cell lysates (data not shown). Our results showed that exposure to AITC significantly decreased protein expression levels of HDAC6, HDAC4, CBP and acetyl CBP/p300 only (Fig. 3a). Then, we focused on the acetylation patterns of specific lysine residues on the tails of both H3 and H4. To this end, total histone extracts of AITC-exposed A375 cells were utilized for the determination of the acetylation profile of H4 on lysines (K) 5, 8, and 12 as well as of H3 on lysines (K) 9, 27, 14, and 18 (data not shown). Diminished expression levels of H4K5Ac, H4K8Ac, and H4K12Ac only were observed when compared to control cells (Fig. 3b). Finally, when total nuclear HDAC and HAT activity levels were evaluated, no statistically significant changes occurred between A375-exposed vs control cells (Fig. 3c).

### Exposure to panobinostat and anacardic acid influences the effect of AITC exposure on the expression levels of acetylated H4 on specific lysine residues K5, K8 and K12

**Fig. 4:**
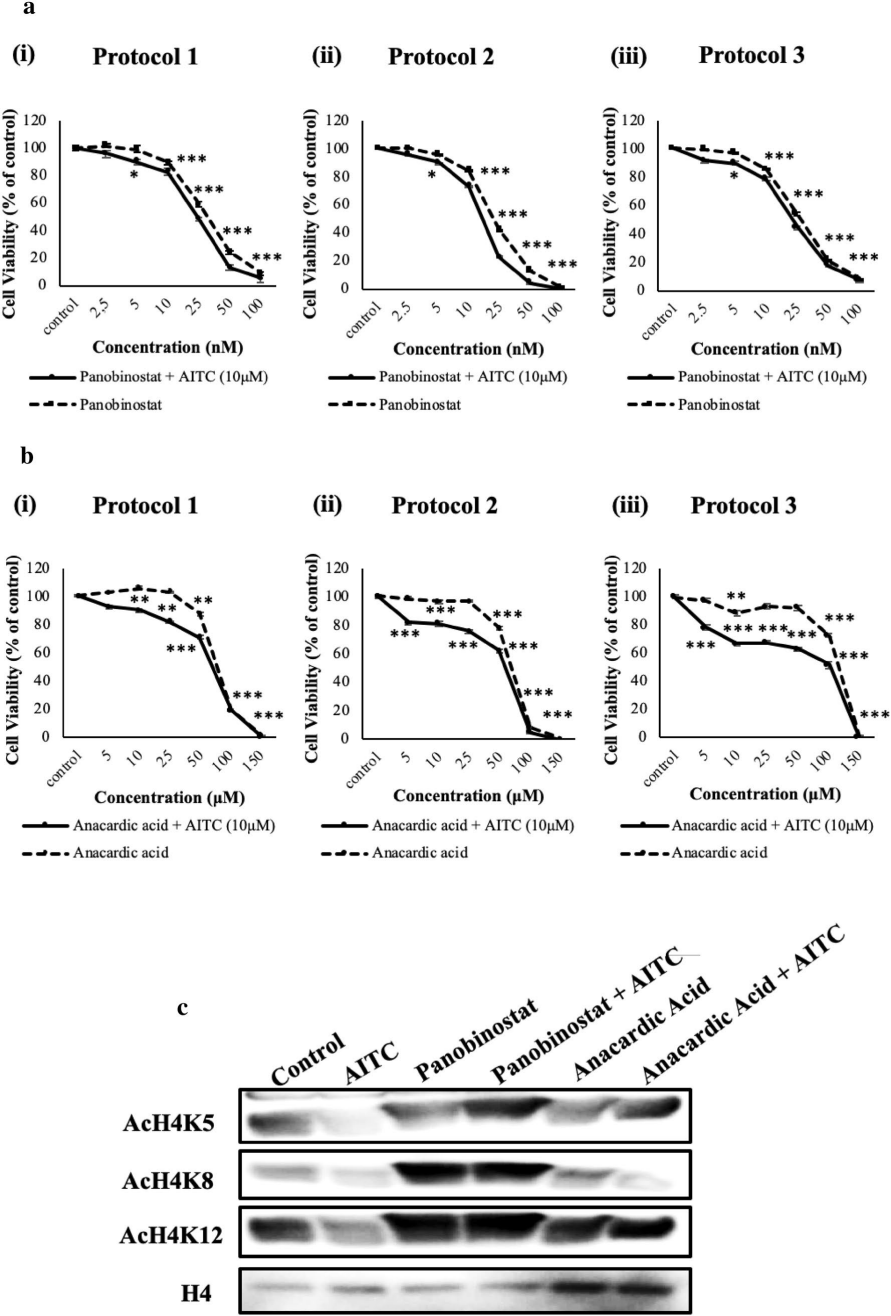
The effect of panobinostat and anacardic acid on histone acetylation, deacetylation and specific H4 lysine acetylation marks in human malignant melanoma (A375) cells. A375 cells were exposed to AITC (10 µM) and either panobinostat (**a**) or anacardic acid (**b**) under three experimental protocols (described in “Materials and methods”): **ai, bi** Protocol 1, **aii, bii** Protocol 2 and **aiii, biii** Protocol 3. Data are expressed as means ± SEM and are representative of three independent experiments. Statistical significance was set at *p < 0.05, **p < 0.01, ***p < 0.001 relative to corresponding controls; **c** A375 cells were co-exposed to AITC (10 µΜ) and either panobinostat (10 nM) or anacardic acid (50 µΜ) under Protocol 2 (described in “Materials and methods”). Western immunoblotting was used, in histone extracts, in order to assess the expression levels of AcH4K5, AcH4K8 and AcH4K12

In an attempt to further investigate if the observed reduction on histone acetylation status depends on decreased activity of HATs, rather than of HDACs, we utilized either panobinostat (an HDAC inhibitor) or anacardic acid (a HAT inhibitor) under the three above-mentioned exposure protocols, in A375 cells. Our data revealed that all three protocols utilizing panobinostat (2.5–10 nM) showed no significant changes on viability of A375 cells when compared to AITC alone. Furthermore, panobinostat was shown to be cytotoxic at 10 nM onwards (Fig. 4ai–iii). On the other hand, utilization of anacardic acid resulted in increased viability when compared to AITC alone [under all three experimental protocols (5–50 µM)] while at 50µΜ onwards there was also significant cytotoxicity observed (Fig. 4bi–iii). Moreover, we determined specific alterations in acetylation levels of H4 following inhibition of HDACs or HATs by means of western immunoblotting. More specifically, a combination of AITC (10µΜ) with panobinostat (10 nM) (under protocol 2) led to an increase in the acetylation status of H4K5, H4K8 and H4K12. On the contrary, co-exposure of A375 cells with AITC (10 µΜ) and anacardic acid (50 µΜ) (under protocol 2) abrogated the effect of AITC on the de-acetylation status of H4K5, H4K8 and H4K12 (Fig. 4c).

### AITC inhibits protein expression levels of HMTs in addition to specific histone H3 lysine methylation marks in human malignant melanoma (A375) cells

**Fig. 5:**
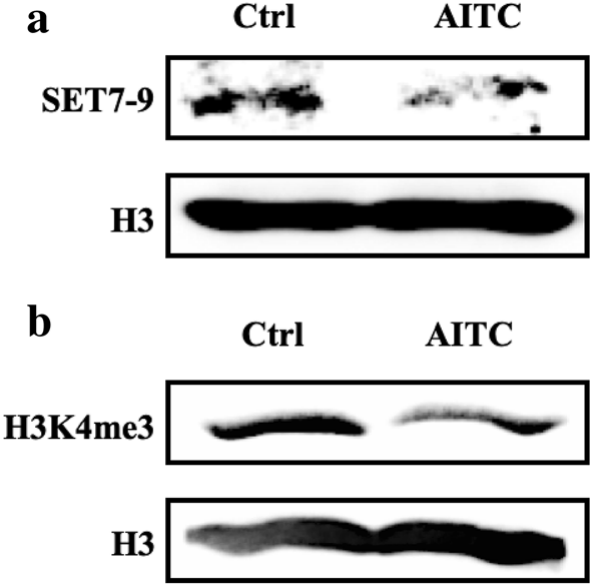
The effect of AITC on histone methylation status and specific H4 lysine methylation marks in human malignant melanoma (A375) cells. A375 cells were exposed to AITC (10 µM) for 48 h. Western immunoblotting was used, in nuclear and histone extracts, in order to assess the expression levels of **a** SET7-9 and **b** tri-methylation status of lysine (K)4 on histone H3

Western blotting on nuclear cell lysates against G9a/EHMT2, RBBP5, ASH2L, SET 8, and SET 7–9 HMTs were utilized (data not shown). Of those, only the expression levels of SET7-9 were significantly diminished in A375-treated cells (Fig. 5a). Next, we determined the effect of AITC on the di- and tri-methylation levels of lysines 4, 9, 27, 36 and 79 on histone H3 (data not shown). Overall, it was shown that exposure to AITC significantly reduced the tri-methylation levels of H3K4me3 only (Fig. 5b).

### AITC induces a differential apoptotic response in human malignant melanoma (A375) cells

In order to examine if the cytotoxic effect of AITC was associated with alterations in apoptotic gene expression, we utilized a genomic approach based on an RT-PCR microarray gene expression profiling methodology. According to our data, there were differences in the induction of various apoptotic genes as a response to AITC exposure in A375 cells. More specifically, intrinsic (e.g., *BAK1, CASP9*), extrinsic (e.g., *FAS, FASLG*) and p53-dependent (*MDM2*) apoptotic genes were shown to be up-regulated. In addition, other apoptotic genes were also shown to be either up-regulated (e.g., *F2RL3, IL2, IL6, PRKCB*) or down-regulated (e.g., *EGFR*) as well (Table 1).

**Table 1:** Apoptotic gene expression in A375 cells exposed to AITC (10 µM) over 48 h

Gene	AITC
*BAK1*	↑ 3.2
*CASP9*	↑ 1.7
*EGFR*	↓ 0.4
*F2RL3*	↑ 15.1
*FAS*	↑ 1.9
*FASLG*	↑ 4.0
*IL2*	↑ 3.2
*IL6*	↑ 3.3
*MDM2*	↑ 3.9
*PRKCB*	↑ 3.0

Data are expressed as fold increase in comparison to control and analyzed by the ΔΔ*C*t method. Observed differences were expressed as fold changes in gene expression by using the DataAssist v3.01 software. (↑) denotes up-regulation, whereas (↓) down-regulation. Data shown are mean values from two independent experiments

## Discussion

The potential of ITCs to prevent melanogenesis has been documented in a number of in vitro [12, 13, 32–37] and in vivo [14, 15, 38–41] studies. Overall, our results showed that exposure to AITC (2.5–50 µM) reduced viability in human A375 and Hs 294T as well as murine B16-F10 melanoma cells in a concentration- and time-dependent manner. In particular, AITC significantly reduced viability of these cells (at 10 µM onwards) while human VMM1, A431 and HaCaT cells remained relatively resistant. Moreover, of all these cell lines, only A375 cells appeared to be the most sensitive to the effect of AITC thereby providing the rationale for their subsequent use. To this end, it was apparent that AITC was capable of modulating the apoptotic response by mediating the differential expression of a number of genes representative of various apoptotic cascades (e.g., intrinsic, extrinsic, p53-dependent apoptosis, etc.) upon exposure to A375 cells.

In the context of regulating gene expression, both acetylation and methylation of histone proteins have been known as important modulators primarily through changes in chromatin structure. Specifically, regarding melanoma pathogenesis, overexpression of class I and II HDACs has been associated with the disease progression and drug resistance [42–45]. On the other hand, ITCs have recently been reported as potent HDAC inhibitors thus disrupting the ratio of HAT/HDAC in a manner capable of inducing cell death in various cancers [46–48]. Furthermore, inhibition of these enzymes has been associated with modulation in the expression of genes involved in tumor suppressor mechanisms including those of Nrf-2-dependent-detoxification of xenobiotics, cell cycle inhibition and apoptosis-induced cancer cell death [17, 18, 21, 49, 50]. Among the genes reported to be regulated by HDAC inhibitors, the re-activation of p21^WAF1/Cip1^ resulting in cell cycle inhibition and apoptosis is the most common one [50–54]. In this study, our data revealed a reduction in protein expression levels of HDACs 4 and 6 but without a significant decrease in total HDAC activity. Similarly, there was a reduction in protein expression levels of CBP and acetyl CBP/p300 but also without an accompanied decrease in the activity levels of HATs, upon AITC exposure. To this end, work by others has shown that inhibition of CBP/p300 promotes cell cycle arrest and cellular senescence, deregulates DNA/damage response and induces apoptosis in melanoma cells [55–57]. Such findings suggest that AITC could act as a potent HAT inhibitor capable of suppressing melanoma cell proliferation. In addition, we evaluated the histone acetylation status on specific lysine residues, at both H3 and H4 N-terminus, and we observed a dramatic decrease on the acetylation levels of lysines 5 (H4K5Ac), 8 (H4K8Ac) and 12 (H4K12Ac) on histone H4. Of these, H4K8 and H4K5 are known to be target sites for the action of CBP/p300 as this HAT is being known to preferentially acetylate these particular lysine residues [58]. In comparison, there were no significant changes associated with the acetylation levels of lysines 9 (H3K9Ac), 14 (H3K14Ac), 18 (H3K18Ac), and 27 (H3K27Ac) of histone H3 upon exposure to AITC (data not shown). Our data, also revealed that combined exposure of AITC with panobinostat (known as an HDAC inhibitor [59–61]) increases the acetylation status of H4K5, H4K8 and H4K12 which, in turn, suggests that inhibition of HDACs could lead to a higher turnover of HATs (perhaps as a compensation mechanism) leading to higher acetylation levels in these lysine residues. Furthermore, co-exposure of AITC with anacardic acid (known as a HAT inhibitor [62–64]) abrogates the effect of AITC on the de-acetylation status of H4K5, H4K8 and H4K12, and, in such case, it restores the acetylation status of these lysine residues back to their control levels. Finally, it is worth mentioning that inhibition of total DNA methylation by decitabine [65, 66] did not show any impact in the context of rescuing A375 cells from the observed AITC-induced cytotoxicity suggesting that such cytotoxicity is not linked to increased DNA methylation.

On another note, the extent of histone methylation (mono-, di-, and tri-) has also been shown to influence the extent of acetylation on H3. It is noteworthy that we have observed SET7-9 to be downregulated in this study. This histone methyltransferase (HMT) enzyme is known to catalyze the mono-methylation of H3K4 and is also associated with the methylation of non-histone proteins including p53. The role of this HMT in carcinogenesis is controversial as some studies report its tumor suppressor function [67, 68] while others associate its activity with increased proliferation [69]. Overall, among all di- and tri-methylated lysines on histone H3 that we examined (K36me2/me3, K4me2/me3, K79me2/me3, K27me2/me3 and K9me2/me3), it was observed that only the expression levels of H3K4me3  were significantly reduced upon exposure to AITC. Specifically, this is an epigenetic modification capable of regulating gene expression by means of activating the transcriptional process. Although one of the least abundant histone modifications, it is used as an epigenetic mark in order to identify active gene promoters [70, 71].

**Fig. 6:**
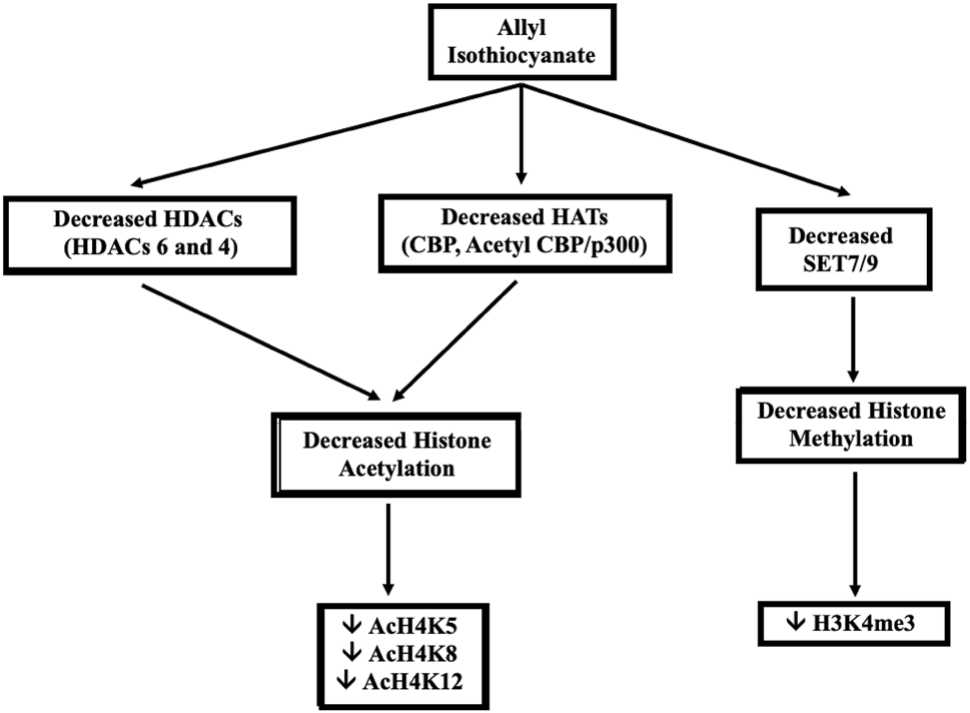
General scheme of the AITC-induced epigenetic response in human malignant melanoma (A375) cells

To conclude, we have shown a significant involvement of AITC in regulating the epigenetic response by modulating specific lysine acetylation(s) and/or methylation(s) on histone proteins H3 and H4 as well as the expression of enzymes capable of catalyzing such epigenetic modifications (Fig. 6). In principle, such a response can impact on  transcriptional activation and/or repression and consequently alter the outcome of gene expression. To our knowledge, this is the first report documenting a detailed characterization of the interaction of AITC with the epigenome, in human malignant melanoma, a finding that highlights the importance of dietary interventions in regulating the epigenome as a result of their action against various types of cancer.
